# Acoustic Correlates of Compensatory Adjustments to the Glottic and Supraglottic Structures in Patients with Unilateral Vocal Fold Paralysis

**DOI:** 10.1155/2015/704121

**Published:** 2015-10-18

**Authors:** Luis M. T. Jesus, Joana Martinez, Andreia Hall, Aníbal Ferreira

**Affiliations:** ^1^Institute of Electronics and Informatics Engineering of Aveiro (IEETA), University of Aveiro, 3810-193 Aveiro, Portugal; ^2^School of Health Sciences (ESSUA), University of Aveiro, 3810-193 Aveiro, Portugal; ^3^Department of Mathematics (DMat), University of Aveiro, 3810-193 Aveiro, Portugal; ^4^Department of Electrical and Computer Engineering, University of Porto, 4200-465 Porto, Portugal

## Abstract

The goal of this study was to analyse perceptually and acoustically the voices of patients with Unilateral Vocal Fold Paralysis (UVFP) and compare them to the voices of normal subjects. These voices were analysed perceptually with the GRBAS scale and acoustically using the following parameters: mean fundamental frequency (*F*0), standard-deviation of *F*0, jitter (ppq5), shimmer (apq11), mean harmonics-to-noise ratio (HNR), mean first (*F*1) and second (*F*2) formants frequency, and standard-deviation of *F*1 and *F*2 frequencies. Statistically significant differences were found in all of the perceptual parameters. Also the jitter, shimmer, HNR, standard-deviation of *F*0, and standard-deviation of the frequency of *F*2 were statistically different between groups, for both genders. In the male data differences were also found in *F*1 and *F*2 frequencies values and in the standard-deviation of the frequency of *F*1. This study allowed the documentation of the alterations resulting from UVFP and addressed the exploration of parameters with limited information for this pathology.

## 1. Introduction

A neural dysfunction of the larynx leads to alterations in voice, respiration, and airway protection. Usually, Unilateral Vocal Fold Paralysis (UVFP) is related to a set of well-documented perceptive alterations such as weak voice, breathiness, roughness, diminished voice intensity, vocal effort, low voice efficiency, voice breaks, diplophonia, and air loss [1–5]. Furthermore, vocal strain is a critical component in various vocal pathologies including UVFP. A neuronal dysphonia, such as UVFP, can alter the vibrational patterns of the Vocal Folds (VF) which leads to compensatory adjustments to the glottic and supraglottic structures that increase the vocal effort and vocal strain perception [6, 7]. In addition to the perceptive alterations, UVFP also results in higher values of jitter and shimmer and lower values of the harmonics-to-noise ratio (HNR) [1–4, 8]. Furthermore, values of standard-deviation of fundamental frequency (*F*0) are reported as higher than normal because of the diminished control of the vibrational pattern of the VF, causing greater variability [9–11]. According to Schwarz et al. [6], there is a need to describe and understand the UVFP patient's larynx configuration for a better and more individualised vocal intervention, preventing compensatory adjustments. Formant frequencies provide acoustic cues about the vocal tract configuration [12–14]. According to Lee et al. [15] the formant's values are relevant for discriminating normal from pathologic voices and the configuration of the vocal tract is different during phonation in people with vocal pathologies. The same authors [15] found slightly lower values of the first formant (*F*1) frequency and higher values of the second formant (*F*2) frequency in cases of UVFP. This indicates that UVFP subjects tend to have a more elevated and advanced tongue position during phonation [13, 14]. A breathy voice (common in UVFP) is reported to be associated with the same configuration referred to previously [16]. However, Titze [13] reports an approximation of the values of the frequency of* F*1 and* F*2 in cases of narrower vocal tract. These vocal tract modifications may result from the attempt to compensate the vocal alteration by patients exhibiting UVFP [2]. According to Lee et al. [15] the standard-deviations of the frequency of* F*1 and* F*2 have higher values in cases of UVFP indicating a higher instability of the vocal tract configuration during phonation.

The aim of this study was to compare perceptually and acoustically the voices of subjects with UVFP and the voices of subjects representing normal quality. Measures related to the vocal tract configuration, namely, formant frequencies, were also analysed and correlated with alterations caused by vocal pathology.

## 2. Materials and Methods

This is a quantitative, descriptive, and cross-sectional study [17–19]. The recordings were made in Hospital de Santo António and Hospital de São João, both in Porto, Portugal, and at the Speech, Language, and Hearing Laboratory (SLHlab) at the University of Aveiro, Portugal. This took place as part of the data collection process of the first representative European Portuguese pathological voice database [20]. Part of this data was divided into two groups: a group having vocal pathology (UVFP) and a group without vocal pathology. A group of 17 patients, evaluated with videolaryngoscopy and diagnosed with UVFP, formed the pathologic group. The inclusion criteria for this group were having diagnosis of UVFP, not having had speech and language therapy intervention, and being over 18 years old. The exclusion criteria were having other concomitant pathologies to UVFP and/or having been submitted to a surgical intervention to correct the vocal pathology. A group of 85 normal voice volunteers were included in the control group based on two distinct procedures: 43 subjects were evaluated with videolaryngoscopy and diagnosed as normal; 42 subjects were evaluated using a vocal anamnesis and summative evaluation (a similar procedure was used by Roark et al. [21]). The inclusion criteria for the control group were having normal voice quality and being over 18 years old. The exclusion criterion was having vocal or other pathologies that may interfere with normal voice production.

Each pathologic case was individually matched to five subjects of the control group in order to increase the power of statistical tests [17, 22]. The cases were matched according to gender and age. The first variable was gender because after puberty there is a set of different characteristics that differentiate male and female voices [23]. The second variable was age because with aging some functional and structural modifications occur at phonatory level [23, 24]. Taking into account the fact that there are notable voice changes if the subjects' age difference is more than 10 years [25–29] the maximum allowed difference of age between the matched subjects was 5 years, in an attempt to reduce variability.

Four (4) subjects with UVFP were male (23.5%) and 13 subjects were female (76.5%). The youngest patient was 30 years old and the oldest 72. The mean age for the pathologic group was 56.7 years with a standard-deviation of 12.7 years. Nine (9) patients had left UVFP (52.9%) and 8 right UVFP (47.1%). In the control group 20 subjects were male (23.5%) and 65 were female (76.5%). The mean age of the control group was 56.1 years and the standard-deviation was 12.7 years.

The voice recordings were made in a clinical setting using* Praat 5.3.56 (32-bit edition)* [30]. A Behringer ECM8000 microphone and a Presonus AudioBox USB (16 bits and 48000 Hz) were used for all of the recordings. The subjects were seated and the microphone was aligned to the mouth at a distance of 30 cm [31, 32]. An informed consent was signed and the vowel [a] was recorded. A parcel of the vowel was then annotated according to criteria defined by Pinho et al. [3]: 200 ms after the onset of phonation and with approximately 100 cycles. This parcel was then manually analysed with* Praat 5.3.56 (64-bit edition)* with an autocorrelation method (used by default by the software) to estimate* F*0. There were some errors in the identification of the period, so a modification of the “octave cost” to a higher value was made (as suggested in* Praat*'s manual). The values of the parameters used to run the autocorrelation method are presented in Table 1.

From the “voice report”* Praat* window the following values were extracted: mean* F*0; standard-deviation of* F*0; jitter (ppq5); shimmer (apq11); mean harmonics-to-noise ratio (HNR). The Burg [33] method (used by default by* Praat*) was used to track the formants. The “formant listing” for the same 100 cycles was obtained and the mean value and standard-deviation were calculated for the frequency of* F*1 and* F*2. The values were double-checked through the spectrogram of each segment.

Each voice was also perceptually assessed using the GRBAS scale [34]. For the pathologic voices a group of five speech and language therapists with expertise in voice assessment made the perceptive evaluation. For the normal voices one speech and language therapist made the perceptive assessment. For these procedures the experts used the following headphones connected to the internal soundcard of a laptop computer: Sennheiser HD 380 Pro; Sennheiser HD201; Sony MDR-CD270; Sony MDRZX100B; Sony MDR-ZX110NA. All of the assessments were made blindly regarding the group (patients or normal subjects).

For the statistical analysis* IBM SPSS Statistics version 20* was used. The interrater consistency was analysed using the Kendall *W* Coefficient. The Mann-Whitney *U* test was used to analyse the GRBAS scale parameters. The acoustic parameters that had normal distribution (HNR,* F*2♀, standard-deviation of* F*0♂,* F*1♂) were statistically analysed using the *t*-test and parameters that did not have normal distribution (*Jitter* (ppq5),* Shimmer* (apq11),* F*0♀, standard-deviation of* F*0♀,* F*1♀, standard-deviation of* F*1♀, standard-deviation of* F*2♀,* F*0♂, standard-deviation of* F*1♂,* F*2♂, and standard-deviation of* F*2♂) were analysed with the Mann-Whitney *U* test. The normality was tested with the Shapiro-Wilk test. A level of significance of 0.05 was used for all statistical analyses.

All of the procedures had the acceptance of the Ethical Commission of the Hospital de Santo António and Hospital de São João. An authorisation from the National Commission for Data Protection was also obtained.

## 3. Results and Discussion

### 3.1. Interrater Consistency

The consistency between the five judges that assessed the pathologic voices was analysed using Kendall's *W* test.  Table 2 shows that there is consistency in all of the parameters of the GRBAS scale between judges. The fact that the judges presented consistency between them indicates that they have a similar internal understanding of the used instrument [35]. This consistency is likely to be related to the fact that the GRBAS scale is widely used, understood, and recommended worldwide by clinicians [36]. The *W*'s value, shown in Table 2, can vary between 0 (no general tendency of consistency between judges) and 1 (all judges responded equally) [37]. In Table 2 we can also see that the lowest value of *W* was found for the R (Rough) parameter. This may be due to the fact that this parameter is a supraclass of perceptive parameters that can lead to various interpretations between different judges [38]. The fact that none of the parameters had a very good consistency was expected because the perceptive assessment is a very complex procedure that includes various subjective elements that are not totally understood [36, 39]. Despite the results varying from* reasonable* to* good*, perceptive evaluation is still a central procedure in the vocal assessment [40].

### 3.2. Comparison of GRBAS Scale Parameters between Normal and UVFP Voices

**Figure 1:**
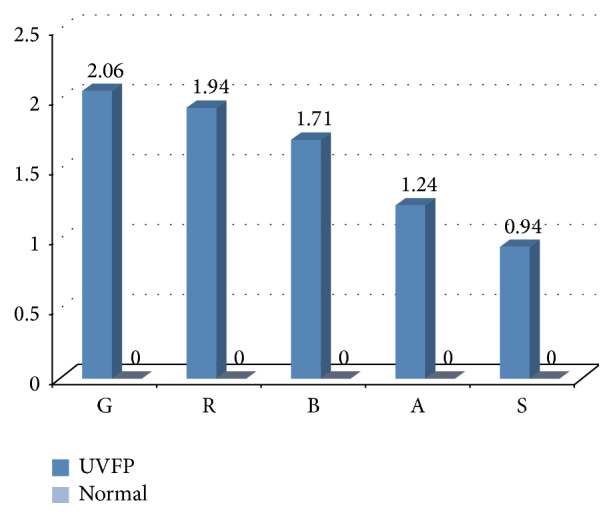
Comparison between mean scores of the GRBAS scale for normal and UVFP subjects.

The results of the perceptive assessment of the voices of the normal and UVFP subjects were analysed using the Mann-Whitney *U* test.  Table 3 shows that all of the GRBAS parameters were statistically different between groups, being higher in the pathologic group as expected (see Figure 1). The control group had a mean score of zero, which was expected because the control group was intended to have a normal/nonaltered voice quality that would be associated to a 0 value (normal) of all parameters assessed in GRBAS. In the pathologic group we can see that the parameter with the highest values was G (Grade), which has been observed before by other authors [41, 42]. In this group of UVFP there were alterations in all of the GRBAS parameters, varying between a mild and moderate grade of perturbation. Grade (G), Rough (R), and Breathy (B) presented the highest mean scores, as previously observed by various authors [1–4, 43]. Another disturbance that is commonly found in subjects with UVFP is a weak voice [1, 4, 44] which is reflected in parameter A (Asthenic), also found in this sample. In addition to the previous parameters, according to Rosenthal et al. [7], it is usual to find vocal strain (parameter S) in these cases, which could also be observed in this study.

One of the major alterations caused by UVFP is the incomplete glottal closure that originates excess air during phonation that creates a breathy voice (parameter B is altered) [2, 4, 45]. This air leakage leads to a lower voice energy originating a weak voice (parameter A is altered) [2, 4, 45]. The irregularity of the VF cycles (parameter R reflects this) is due to the reduced mobility/immobility of the paralysed VF or to the fact that the unhealthy VF may present a passive vibration [4, 46]. In some cases, in an attempt to overcome the alterations caused by the UVFP, patients create compensations that can lead to strain in the supraglottic region, increasing the vocal effort and giving the voice a strained characteristic (parameter S) [4, 7]. Grade (G) is related with the other parameters and varies according to the severity of the overall voice perturbation [47].

### 3.3. Comparison of Acoustic Parameters between Normal and UVFP Voices

**Figure 2:**
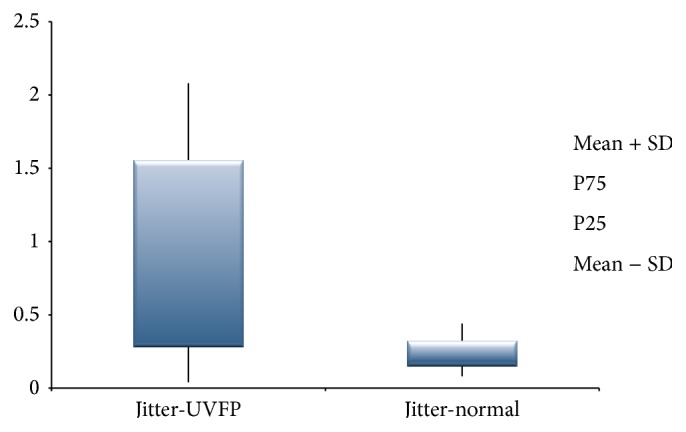
Jitter (%) values for UVFP and normal subjects.

**Figure 3:**
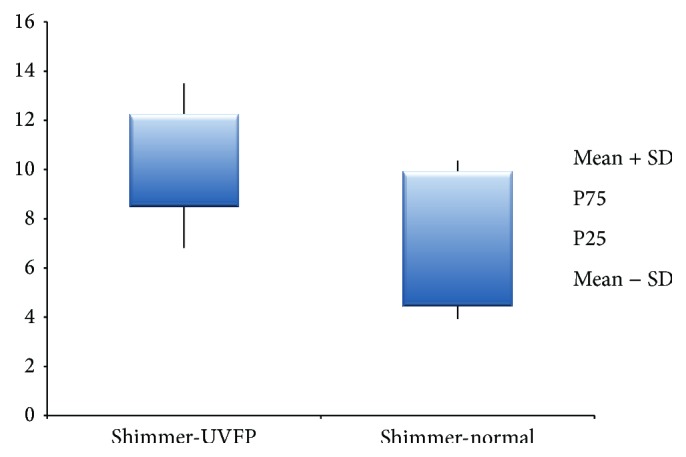
Shimmer (%) values for UVFP and normal subjects.

**Figure 4:**
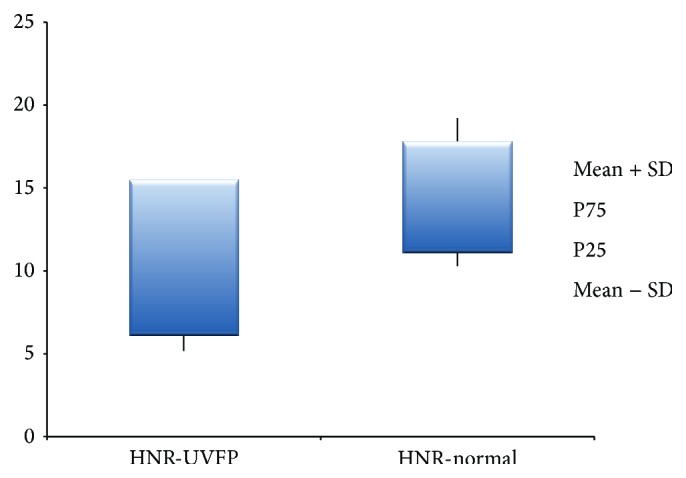
HNR (dB) values for UVFP and normal subjects.

Although perceptive assessment is the most used technique for vocal assessment, it is a subjective process that leads to some variability issues [8]. Contrary to this, acoustic data allows objective and noninvasive measures about the behaviour of the VF [8, 15, 48–50].  Table 4 shows statistically different values of jitter (ppq5), shimmer (apq11), and HNR between the normal and pathologic voices. Jitter, which is related to the absolute difference between the durations of consecutive cycles [43], is higher in UVFP subjects (see Figure 2). These results were also obtained by other authors [2, 3, 8]. These higher values may be due to the asymmetry at the VF level, caused by the UVFP that leads to vibration irregularities in frequency altering the jitter values [2]. Similarly shimmer, which is related to the absolute difference between the amplitudes of consecutive cycles [43], is also higher in UVFP cases (see Figure 3). These results were also obtained by other authors [2, 3, 8]. The asymmetry caused by UVFP leads to vibration irregularities in amplitude altering shimmer values [2]. This parameter is also increased by a poor and inconsistent contact between VF, which is very common in UVFP [51]. Thus, these UVFP subjects present more cyclic irregularity at frequency and amplitude level compared to the normal voice subjects. It should be noted that we also had higher than normal values of shimmer in the normal sample. This may be due to the fact that the recordings were made in a clinical setting that is not entirely noise-free and this could have interfered with the data calculation of this parameter. Regarding the HNR, which is obtained from the ratio between the harmonic and noise components of the signal [43], the results indicate a lower value in the pathologic group (see Figure 4). These results were consistent with the literature [2, 8]. The alterations in periodicity caused by the UVFP originate a lower ratio between the two components, diminishing the HNR values in the pathologic cases [2]. These results indicate that patients with UFVP have higher relative noise amplitude during phonation (than the normal subjects) lowering the HNR value.

**Figure 5:**
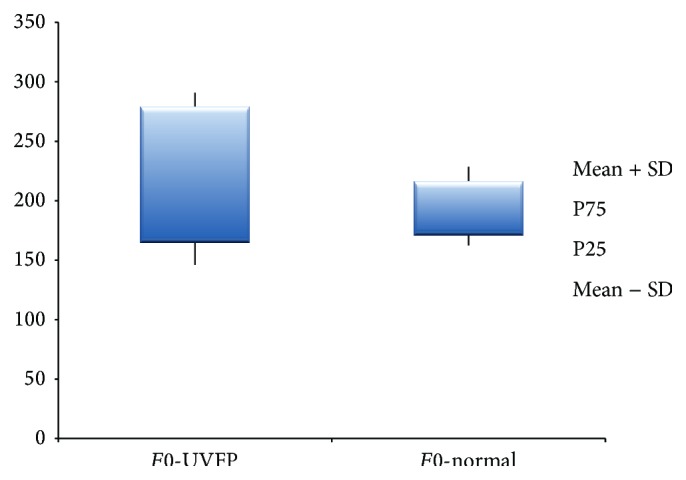
*F*0 (Hz) values for female UVFP and normal subjects.

**Figure 6:**
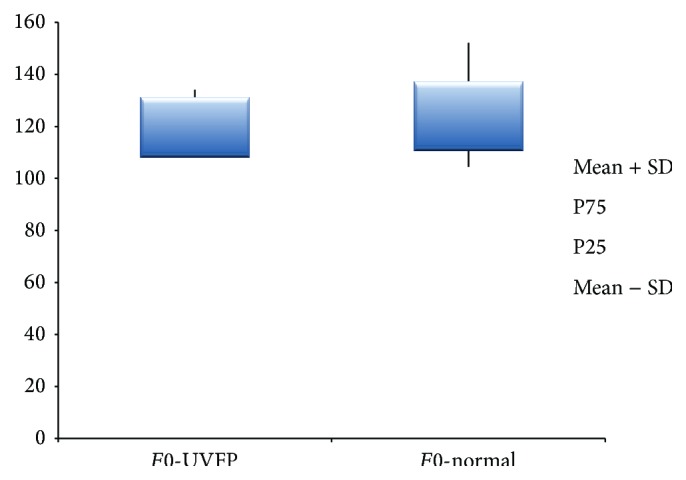
*F*0 (Hz) values for male UVFP and normal subjects.

The parameters presented in Tables 5 and 6 were divided by gender because females and males have different inherent vocal tract and VF characteristics, especially in terms of size and mass [52]. For* F*0 (see Figures 5 and 6) we can see that there are no significant statistical differences between pathologic and normal voices in both genders. This fact was also previously described by Oguz et al. [8]. Fundamental frequency is directly related to and dependent of length, tension, mass, rigidity, and the interaction with the subglottic pressure [53]. The fact that there are no differences between the two groups indicates that, in this sample, the modifications at VF level caused by UVFP are not sufficient to create real alterations in* F*0. Also according to Woo et al. [54] the majority of UVFP subjects present* F*0 values close to normal.

**Figure 7:**
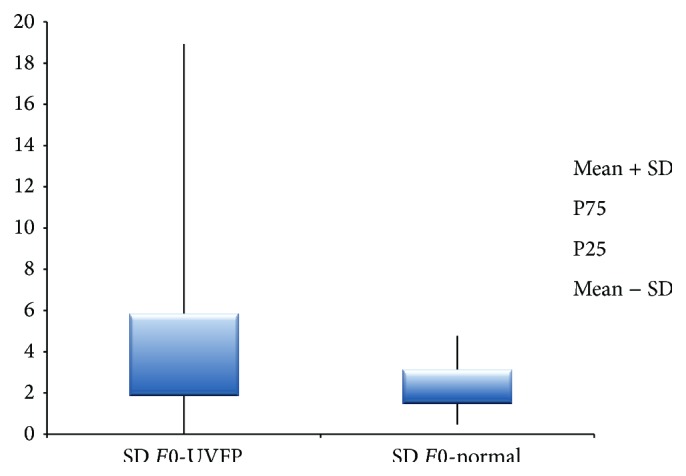
SD of* F*0 (Hz) values for female UVFP and normal subjects.

**Figure 8:**
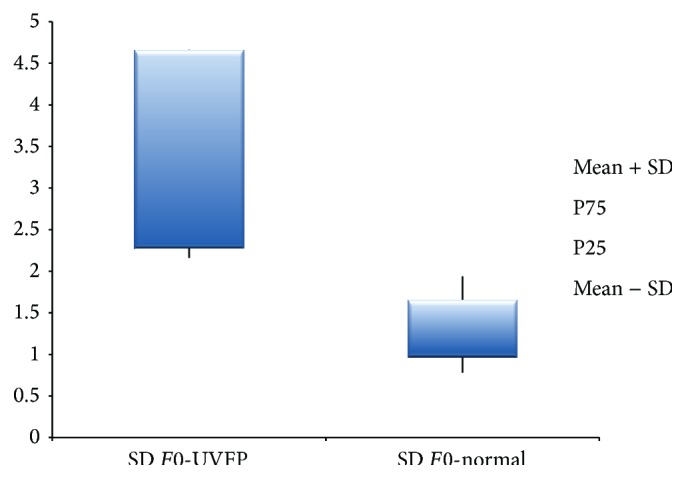
SD of* F*0 (Hz) values for male UVFP and normal subjects.

The standard-deviation of* F*0 (see Figures 7 and 8), which is related to the variations in vibration and muscular control of the VF, is higher in the pathologic group indicating important alterations in the described aspects [53]. Thus, subjects with UVFP present more* F*0 variability indicating a poorer muscular control and lower vibrational stability of VF. These results are supported by other authors [10, 11, 46, 53].

The vocal tract configuration interacts with VF oscillation; that is, vocal tract configuration constrains VF functioning during phonation [15, 55]. After the onset of UVFP patients usually develop some compensatory adjustments at glottic and supraglottic level altering voice and vocal tract configuration [6]. The description of vocal tract configurations in subjects with UVFP could guide treatments and help prevent negative compensations [6, 7].

**Figure 9:**
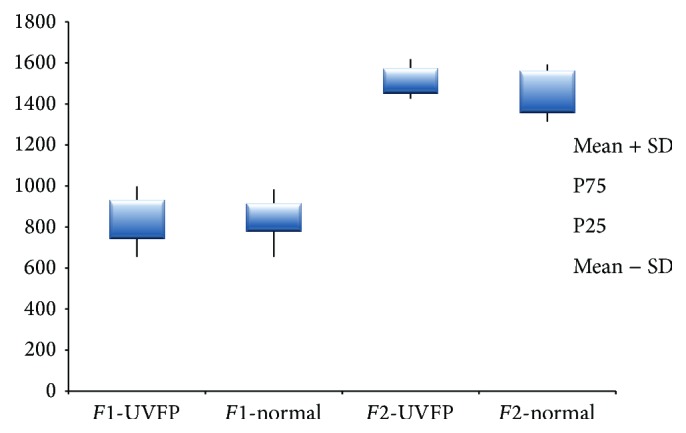
*F*1 and* F*2 frequency (Hz) values for female UVFP and normal subjects.

**Figure 10:**
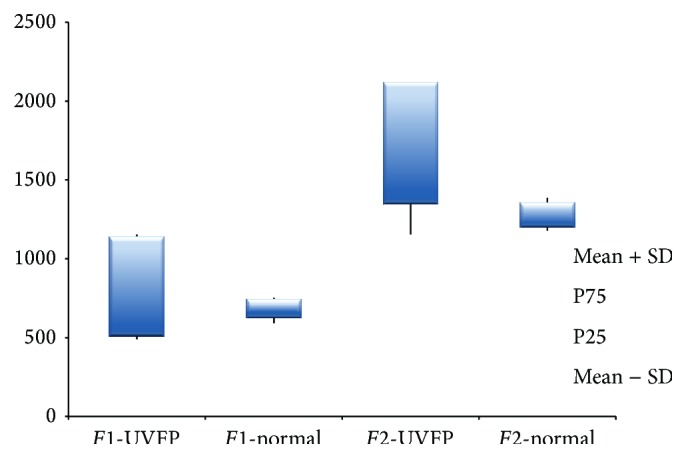
*F*1 and* F*2 frequency (Hz) values for male UVFP and normal subjects.

Regarding* F*1 frequency, Table 5 shows that for females there are no statistically significant differences between pathologic and normal subjects (see Figures 9 and 10). A similar result was obtained by Lee et al. [15]. Formant frequency values shown in Table 6 reveal that, for males, differences between groups are statistically significant. Lower values of* F*1 frequencies in UVFP cases were expected (based on data reported previously [15]); however, Table 6 clearly shows that the* F*1 frequency values were higher in the pathologic group. However, authors such as Hartl et al. [2] and D. H. Klatt and L. C. Klatt [56] have also reported higher* F*1 frequency values for voices with similar characteristics to UVFP patients. Since the frequency of* F*1 is inversely related to the vertical movement of the tongue, higher values of this formant (in UVFP subjects) indicate a lower tongue position during phonation for the pathologic subjects. This result is in line with what was found by Higashikawa et al. [57] for whispered voices.

The second formant (*F*2) frequency, which is related to the horizontal tongue movement, is higher in UVFP male subjects (see Figures 9 and 10). This result was also obtained in other studies [2, 15]. For females, although the *p* value is very close to the significance level, there are no statistically significant differences between normal and UVFP subjects. However, we can see a slightly higher value of* F*2 frequency in the female pathologic group compared to normal females. Therefore results indicate that there could be a tendency to a more advanced tongue position during phonation in cases of UVFP. This is consistent with the results presented by Lotto et al. [16] who studied breathy voices (typical of UVFP).

**Figure 11:**
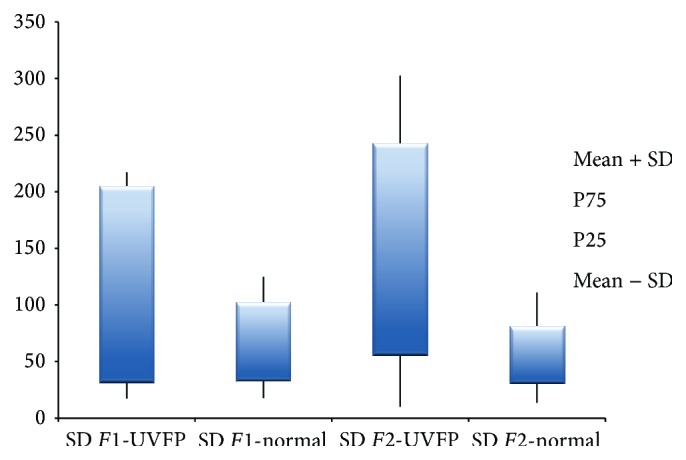
SD of* F*1 and* F*2 frequency (Hz) for female UVFP and normal subjects.

**Figure 12:**
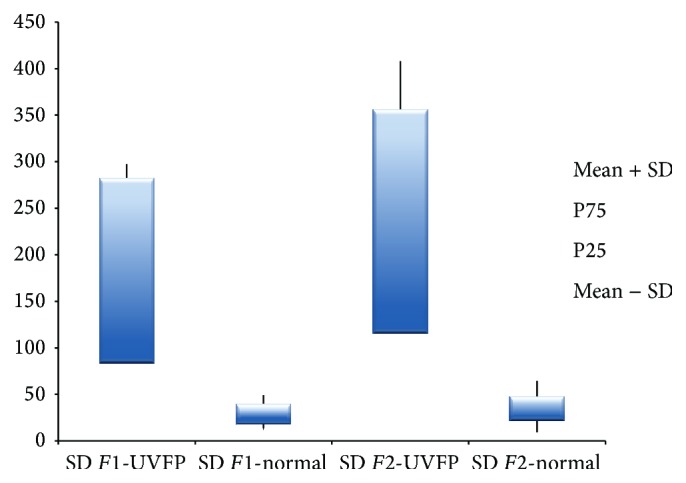
SD of* F*1 and* F*2 frequency (Hz) for male UVFP and normal subjects.

As for the SD of the frequency of* F*1 shown in Table 5, there were significant differences between the two groups being SD of* F*1 frequency higher in the patients, for male participants. There were no significant differences between groups for females (see Table 6). As for the SD of the frequency of* F*2 there were statistically significant higher values in the UVFP group for both genders (see Figures 11 and 12). Therefore these parameters, especially the SD of the frequency of* F*2, may have an important role in discriminating normal and UVFP voices. Pathologic voices showed higher values of formant frequency SD. These results were also obtained by Lee et al. [15]. This indicates a greater instability of the vocal tract configuration in UVFP during phonation.

Overall results related to the vocal tract configuration (*F*1 and* F*2) show great potential to discriminate between normal and UVFP voices (especially for males) in spite of the localisation of the lesion being at the VF level. This is in agreement with the literature which clearly indicates that the behaviour of the VF is not entirely independent of the vocal tract [55, 58, 59]. Thus, these parameters can add useful information to the assessment procedure and may be used as a complement to the more traditional VF behavioural assessment.

It should be noted that the overall results obtained for females distance themselves from what was initially expected. These differences between genders may be due to a greater technical difficulty in analysing female voices [56, 60]. To a large extent, these difficulties are associated with the identification of formants, due to the fact that* F*0 is higher, and this increases the difficulty in* F*1 estimation [56].

## 4. Conclusions

In this study various ways of assessing the UVFP voice were combined. Since vocal therapy is one of the first noninvasive treatment options with potential to help the client to reacquire a functional voice, it is fundamental to know in detail the alterations created by the pathology at VF and vocal tract level to better guide the treatment. Perceptual differences between normal and UVFP voices were found. The perceptual parameters that better characterised this data of UVFP subjects were Rough (R) and Breathy (B), but altered values of Asthenic (A) and Strained (S) were also found. As far as acoustic parameters are concerned there were no differences in* F*0 values between normal and UVFP voices in this sample. Jitter (ppq5), shimmer (apq11), HNR, and SD of* F*0 had an important role in discriminating normal and UVFP voices. Measures related to the vocal tract configuration were also indicative of alterations at VF level; therefore the analysis of formant frequencies values and their SD may have an important role in a clinical setting contributing to a better knowledge of the alterations caused by the vocal pathology. Future work should continue to explore formants and their relation to vocal pathology.

## Figures and Tables

**Table 1:** Values of the autocorrelation method used in *Praat* for the voice analysis.

Parameter	Value
Maximum number of candidates	15
Silence threshold	0.03
Voicing threshold	0.45
Octave cost	0.15
Octave-jump cost	0.35
Voiced/unvoiced cost	0.14

**Table 2:** Interrater consistency—Kendall's *W* test.

Scale parameter	*W*	*p* value
G	0.263	0.001
R	0.160	0.033
B	0.381	<0.001
A	0.344	<0.001
S	0.438	<0.001

G: Grade; R: Rough; B: Breathy; A: Asthenic; S: Strained; *W*: Kendall's *W*.

**Table 3:** Comparison of the results of GRBAS scale between UVFP and normal voice subjects.

	UVFP		Normal		*U*	*p* value
	*N*	Mean ± SD	*N*	Mean ± SD		
G	17	2.06 ± 0.827	85	0	0	<0.001
R	17	1.94 ± 0.899	85	0	0	<0.001
B	17	1.71 ± 0.772	85	0	0	<0.001
A	17	1.24 ± 0.437	85	0	0	<0.001
S	17	0.94 ± 0.556	85	0	25.5	<0.001

UVFP: Unilateral Vocal Fold Paralysis; G: Grade; R: Rough; B: Breathy; A: Asthenic; S: Strained; *N*: number of cases; SD: standard deviation; *U*: Mann-Whitney *U* test.

**Table 4:** Comparison of jitter, shimmer, and HNR between normal and UVFP subjects.

	UVFP		Normal		*t* or *U*	*p* value
	*N*	Mean ± SD	*N*	Mean ± SD		
Jitter ppq5 (%)	17	1.06 ± 1.02	85	0.26 ± 0.18	*U* = 249	<0.001
Shimmer apq11 (%)	17	10.16 ± 3.34	85	7.14 ± 3.22	*U* = 376	0.001
HNR (dB)	17	10.11 ± 4.94	85	14.75 ± 4.46	*t* = −3.85	<0.001

UVFP: Unilateral Vocal Fold Paralysis; SD: standard deviation; *t*: *t*-test;  U: Mann-Whitney *U* test.

**Table 5:** Fundamental frequency (*F*0) and first and second formant frequencies (*F*1 and *F*2) and their standard-deviations, for normal and UFVP female participants.

♀	UVFP		Normal		*t* or *U*	*p* value
	*N*	Mean ± SD	*N*	Mean ± SD		
*F*0 (Hz)	13	218.38 ± 72.36	65	195.36 ± 33.02	*U* = 394	0.335
SD *F*0 (Hz)	13	6.65 ± 12.28	65	2.62 ± 2.15	*U* = 267	0.018
*F*1 (Hz)	13	826.16 ± 171.73	65	819.03 ± 164.75	*U* = 421	0.495
SD *F*1 (Hz)	13	117.18 ± 99.91	65	71.28 ± 53.54	*U* = 327	0.116
*F*2 (Hz)	13	1522.69 ± 96.00	65	1453.51 ± 139.20	*t* = 1.58	0.059
SD *F*2 (Hz)	13	156.31 ± 146.20	65	62.26 ± 48.67	*U* = 204	0.002

UVFP: Unilateral Vocal Fold Paralysis; *N*: number of cases; SD: standard deviation; *t*: *t*-test;  *U*: Mann-Whitney *U* test; *F*0: fundamental frequency; SD *F*0: standard-deviation of the fundamental frequency; *F*1: first formant frequency; SD *F*1: standard-deviation of first formant frequency; *F*2: second formant frequency; SD *F*2: standard-deviation of second formant frequency.

**Table 6:** Fundamental frequency (*F*0) and first and second formant frequencies (*F*1 and *F*2) and their standard-deviations, for normal and UFVP male participants.

♂	UVFP		Normal		*t* or *U*	*p* value
	*N*	Mean ± SD	*N*	Mean ± SD		
*F*0 (Hz)	4	121.43 ± 12.70	20	128.27 ± 23.85	*U* = 39	0.485
SD *F*0 (Hz)	4	3.41 ± 1.25	20	1.36 ± 0.58	*t* = 5.27	<0.001
*F*1 (Hz)	4	821.48 ± 331.80	20	677.33 ± 84.95	*t* = 1.81	0.043
SD *F*1 (Hz)	4	191.91 ± 105.62	20	30.58 ± 18.84	*U* = 1	<0.001
*F*2 (Hz)	4	1629.09 ± 474.06	20	1282.95 ± 104.35	*U* = 11	0.011
SD *F*2 (Hz)	4	263.68 ± 144.45	20	36.89 ± 27.53	*U* = 3	0.001

UVFP: Unilateral Vocal Fold Paralysis; *N*: number of cases; SD: standard deviation; *t*: *t*-test; *U*: Mann-Whitney *U* test; *F*0: fundamental frequency; SD *F*0: standard-deviation of the fundamental frequency; *F*1: first formant frequency; SD *F*1: standard-deviation of first formant frequency; *F*2: second formant frequency; SD *F*2: standard-deviation of second formant frequency.

## References

[1] Blitzen A., Brin M., Ramig L. (2009). *Neurologic Disorders of the Larynx*.

[2] Hartl D. M., Hans S., Vaissière J., Riquet M., Brasnu D. F. (2001). Objective voice quality analysis before and after onset of unilateral vocal fold paralysis. *Journal of Voice*.

[3] Pinho C. M. R., Jesus L. M. T., Barney A. (2013). Aerodynamic measures of speech in unilateral vocal fold paralysis (UVFP) patients. *Logopedics Phoniatrics Vocology*.

[4] Sulica L., Blitzer A. (2006). *Vocal Fold Paralysis*.

[5] Verdolini K., Rosen C., Branski R. (2006). *Classification Manual for Voice Disorders-I*.

[6] Schwarz K., Cielo C. A., Steffen N., Becker J., Jotz G. P. (2011). Voice and laryngeal configuration of men with unilateral vocal fold paralysis before and after medialization. *Journal of Voice*.

[7] Rosenthal A. L., Lowell S. Y., Colton R. H. (2014). Aerodynamic and acoustic features of vocal effort. *Journal of Voice*.

[8] Oguz H., Demirci M., Safak M. A., Arslan N., Islam A., Kargin S. (2007). Effects of unilateral vocal cord paralysis on objective voice measures obtained by Praat. *European Archives of Oto-Rhino-Laryngology*.

[9] Chhetri D. K., Neubauer J., Bergeron J. L., Sofer E., Peng K. A., Jamal N. (2013). Effects of asymmetric superior laryngeal nerve stimulation on glottic posture, acoustics, vibration. *Laryngoscope*.

[10] Madill C., McCabe P. (2011). Acoustic analysis using freeware: praat. *Handbook of Voice Assessments*.

[11] Vogel A. (2011). Multidimensional analysis of voice: computerized speech lab. *Handbook of Voice Assessments*.

[12] Alku P. (2011). Glottal inverse filtering analysis of human voice production—a review of estimation and parameterization methods of the glottal excitation and their applications. *Sadhana*.

[13] Titze I. (2000). *Principles of Voice Production*.

[14] Fant G. (1970). *Acoustic Theory of Speech Production—With Calculations Based on X-Ray Studies of Russian Articulations*.

[15] Lee J.-W., Kang H.-G., Choi J.-Y., Son Y.-I. (2013). An investigation of vocal tract characteristics for acoustic discrimination of pathological voices. *BioMed Research International*.

[16] Lotto A. J., Holt L. L., Kluender K. R. (1997). Effect of voice quality on perceived height of English vowels. *Phonetica*.

[17] Gordis L. (2004). *Epidemiology*.

[18] Breakwell G., Smith J., Wright D. (2012). *Research Methods in Psychology*.

[19] Kerlinger F., Lee H. (2000). *Foundations of Behavioral Research*.

[20] Jesus L. (2014). University of Aveiro's advanced voice function assessment databases (AVFAD). *Revista de Saúde Pública*.

[21] Roark R. M., Watson B. C., Baken R. J., Brown D. J., Thomas J. M. (2012). Measures of vocal attack time for healthy young adults. *Journal of Voice*.

[22] Breslow N., Day N. (1980). *Statistical Methods in Cancer Research: Vol.1—The Analysis of Case-Control Studies*.

[23] Beck J., Hardcastle W., Laver J., Gibbon F. (2010). Organic variation of the vocal apparatus. *The Handbook of Phonetic Sciences*.

[24] Linville S., Kent R., Ball M. (2000). The aging voice. *Voice Quality Measurements*.

[25] Chatterjee I., Halder H., Bari S., Kumar S., Roychoudhury A., Murthy P. (2011). An analytical study of age and gender effects on voice range profile in bengali adult speakers using phonetogram. *International Journal of Phonosurgery and Laryngology*.

[26] Fisher H. B., Linville S. E. (1985). Acoustic characteristics of women's voices with advancing age. *Journals of Gerontology*.

[27] Perrin E., Berger-Vachon C., Collet L. (1999). Acoustical recognition of laryngeal pathology: a comparison of two strategies based on sets of features. *Medical and Biological Engineering and Computing*.

[28] Ramig L. A., Ringel R. L. (1983). Effects of physiological aging on selected acoustic characteristics of voice. *Journal of Speech and Hearing Research*.

[29] Shipp T., Hollien H. (1969). Perception of the aging male voice. *Journal of Speech and Hearing Research*.

[30] Boersma P. (2001). Praat, a system for doing phonetics by computer. *Glot International*.

[31] Boyanov B., Hadjitodorov S. (1997). Acoustic analysis of pathological voices: a voice analysis system for the screening and laryngeal diseases. *IEEE Engineering in Medicine and Biology Magazine*.

[32] Švec J. G., Granqvist S. (2010). Guidelines for selecting microphones for human voice production research. *American Journal of Speech-Language Pathology*.

[33] Burg J. Maximum entropy spectral analysis.

[34] Hirano M. (1981). *Clinical Examination of Voice*.

[35] Artstein R., Poesio M. (2008). Inter-coder agreement for computational linguistics. *Computational Linguistics*.

[36] Jalalinajafabadi F., Gadepalli C., Ascott F., Homer J., Lujan M., Cheetham B. Perceptual evaluation of voice quality and its correlation with acoustic measurement.

[37] Kendall M. G., Smith B. B. (1939). The problem of *m* rankings. *Annals of Mathematical Statistics*.

[38] Moers C., Möbius B., Rosanowski F., Nöth E., Eysholdt U., Haderlein T. (2012). Vowel- and text-based cepstral analysis of chronic hoarseness. *Journal of Voice*.

[39] Sellarsa C., Stantona A., McConnachiea A. (2009). Reliability of perceptions of voice quality: evidence from a problem asthma clinic population. *The Journal of Laryngology & Otology*.

[40] Karnell M. P., Melton S. D., Childes J. M., Coleman T. C., Dailey S. A., Hoffman H. T. (2007). Reliability of clinician-based (GRBAS and CAPE-V) and patient-based (V-RQOL and IPVI) documentation of voice disorders. *Journal of Voice*.

[41] Hirano M., Hibi S., Terasawa R., Fujiu M. (1986). Relationship between aerodynamic, vibratory, acoustic and psychoacoustic correlates in dysphonia. *Journal of Phonetics*.

[42] Yu P., Garrel R., Nicollas R., Ouaknine M., Giovanni A. (2007). Objective voice analysis in dysphonic patients: new data including nonlinear measurements. *Folia Phoniatrica et Logopaedica*.

[43] Little M. A., Costello D. A. E., Harries M. L. (2011). Objective dysphonia quantification in vocal fold paralysis: comparing nonlinear with classical measures. *Journal of Voice*.

[44] Bielamowicz S., Stager S. V. (2006). Diagnosis of unilateral recurrent laryngeal nerve paralysis: laryngeal electromyography, subjective rating scales, acoustic and aerodynamic measures. *Laryngoscope*.

[45] Baylor C., Yorkston K., Strand E., Eadie T., Duffy J. (2005). Measurment of treatment outcome in unilateral vocal fold paralysis: a systematic review. *UVFP Technical Report*.

[46] Pinho S., Tsuji D., Bohadana S. (2006). *Fundamentos em Laringologia e Voz*.

[47] Takahashi H., Japan Society of Logopedics and Phoniatrics (1979). Assessment of auditory impression of dysphonia. *Voice Examination Methods*.

[48] Dejonckere P. H., Bradley P., Clemente P. (2001). A basic protocol for functional assessment of voice pathology, especially for investigating the efficacy of (phonosurgical) treatments and evaluating new assessment techniques: guideline elaborated by the Committee on Phoniatrics of the European Laryngological Society (ELS). *European Archives of Oto-Rhino-Laryngology*.

[49] Yan N., Wang L., Ng M. L. Acoustical analysis of voices produced by Cantonese patients of unilateral vocal fold paralysis acoustical analysis of voices by Cantonese UVFP.

[50] Teles V., Rosinha A. (2008). Acoustic analysis of formants and measures of the sonorous signal disturbance in non-smoker and non-alcoholic women without vocal complaints. *International Archives of Otorhinolaryngology*.

[51] Reijonen P., Lehikoinen-Söderlund S., Rihkanen H. (2002). Results of fascial augmentation in unilateral vocal fold paralysis. *Annals of Otology, Rhinology and Laryngology*.

[52] Childers D. G., Wu K. (1991). Gender recognition from speech. Part II: fine analysis. *Journal of the Acoustical Society of America*.

[53] Behlau M. (2001). *Voz: O Livro do Especialista*.

[54] Woo P., Colton R., Brewer D., Casper J. (1991). Functional staging for vocal cord paralysis. *Otolaryngology—Head and Neck Surgery*.

[55] Kent R., Read C. (2002). *The Acoustic Analysis of Speech*.

[56] Klatt D. H., Klatt L. C. (1990). Analysis, synthesis, and perception of voice quality variations among female and male talkers. *Journal of the Acoustical Society of America*.

[57] Higashikawa M., Nakai K., Sakakura A., Takahashi H. (1996). Perceived pitch of whispered vowels-relationship with formant frequencies: a preliminary study. *Journal of Voice*.

[58] Rothenberg M. (1980). Source-tract acoustic interaction in breathy voice. *Vocal Fold Fisiology—Biomechanics, Acoustic and Phonatory Control*.

[59] Titze I. R., Story B. H. (1997). Acoustic interactions of the voice source with the lower vocal tract. *Journal of the Acoustical Society of America*.

[60] Hanson H. M. (1997). Glottal characteristics of female speakers: acoustic correlates. *Journal of the Acoustical Society of America*.

